# A Comparative In-Vitro Study of the Effectiveness of Several Methods of Sterilizing Endodontic Files

**DOI:** 10.7759/cureus.74473

**Published:** 2024-11-26

**Authors:** Moayad AlBaghdadi, Maysam Khaddam, Asmahan Zainab

**Affiliations:** 1 Faculty of Dentistry, Department of Operative and Endodontics, Tishreen University, Latakia, SYR; 2 Faculty of health and medical technology, Department of Medical Laboratory Technology, Al-Ayen University, Nasiriyah, IRQ; 3 Faculty of Science, Department of Microbiology, Tishreen University, Latakia, SYR

**Keywords:** autoclave, diode laser, endodontic files sterilization, enterococcus faecalis, infection control guidelines

## Abstract

Background

Successful endodontic treatment depends on several elements. The most important of which is the elimination of microorganisms within the root canal system. To achieve this, sterility must be maintained during all steps of the treatment. Therefore, it is necessary to ensure the sterility of the tools used in endodontic treatment, whether new or reused. This study aimed to evaluate the effectiveness of several methods for sterilizing endodontic files by evaluating their ability to eliminate a specific type of bacteria.

Methods

Twenty stainless steel K files were divided into four groups. Files were contaminated with a strain of *Enterococcus faecalis*, and then groups were sterilized using different methods (group A: autoclave; group B: chemical solution; group C: no sterilization; group D: diode laser). After sterilization, files were placed in test tubes containing nutrient broth and then were incubated for 24 hours. After incubation, test tubes were monitored for turbidity, and a sample was taken from each tube to detect bacterial growth on plates containing Mueller-Hinton agar. After 24 hours of incubation, bacterial growth on plates and turbidity in test tubes were registered.

Results

Bacterial growth was registered on groups C and D plates, while no bacterial growth was registered on groups A and B plates. Turbidity was shown in groups C and D test tubes, while no turbidity was shown in groups A and B test tubes.

Conclusions

Within the limitation of this study, diode laser was not able to eliminate the bacteria, and thus cannot be used to sterilize endodontic files. The autoclave and the chemical solution were able to eliminate the specified bacteria.

## Introduction

Infection control is one of the most considered factors that affects the prognosis of dental treatment, due to the potential transmission of diseases in dental clinics, which can put the staff and patients at risk. Inadequate disinfection or sterilization of contaminated dental instruments used for one patient before reusing them for another patient can result in the transmission of pathogens. The transmission of microorganisms from one patient to another via contaminated dental tools and devices was reported by several studies, hence effective measures to control this contamination are needed to reduce disease transmission [1].

Endodontic treatment consists of several essential phases such as isolation of the working area, access to the pulp chamber, cleaning and shaping and finally filling the root canals. Each step requires a series of disposable or Sterilizable instruments. Even disposable instruments require prior sterilization procedures and removing manufacturing residues before being utilized in treatments [2]. All instruments used in endodontic treatment are classified as critical instruments according to Spaulding’s classification since these instruments contact the vascular system within the dental pulp [3].

Sterilization methods used in dentistry include: dry heat sterilization, moist heat sterilization, sterilization with chemicals (cold sterilization), gas sterilization, and sterilization by radiation [4]. Lasers such as CO_2_ laser, Diode laser, Argon laser, and Nd: YAG laser have been proposed to be used for sterilization in many researches and have been tested for this purpose. Laser sterilization depends on the absorption of the laser beam by bacteria, which leads to its elimination [5].

Venkatasubramanian et al. found that CO_2_ laser can be used as a routine method and also as a chair-side method for sterilizing endodontic files in routine clinical practice. Although an autoclave is an effective way to sterilize endodontic files, the time taken for sterilization using an autoclave is relatively large. Lasers can sterilize endodontic instruments effectively and quickly as well. The idea of using lasers for sterilizing files became more logical as the use of lasers has become more common in dental clinics [6].

On the contrary, the study conducted by Al-Jamell et al. showed that autoclave sterilization was superior to laser sterilization and that sterilization with CO_2_ laser was superior to diode laser. They found that laser sterilization is a quick method, but less effective than the autoclave [7]. Ameer et al. also found that an autoclave is the only way to achieve complete sterility, as they compared the effectiveness of sterilization using an autoclave, glutaraldehyde, and diode laser [8].

Seeing that the results of the previous articles were contradictory, this study aimed to test the effectiveness of the mentioned methods in eliminating *Enterococcus faecalis*, considering the role that this bacterium plays in the failure of endodontic treatment.

## Materials and methods

This study was registered by Tishreen University Council, Syria (protocol code: 5317, date: August 2023).

Twenty stainless steel K files (Mani, Japan) were used in this study. All the files were pre-sterilized by autoclave (Nisea Premium, Faro Spa, Italy) for 30 minutes/121°C at 15-pound pressure to eliminate prior contamination. The test microorganism used in this study was a strain of *E. faecalis* obtained from the lap of microorganisms, Science faculty, Tishreen University. The *E. faecalis* strain was inoculated in nutrient broth and placed in an incubator to allow the bacteria to grow at 37°C/24 h.

The pre-sterilized files were placed in test tubes containing bacterial broth and left for 24 hours in an incubator for contamination at 37°C. Then the decontaminated files were divided into four groups (five files in each group): Group A: Files in this group were autoclaved for 30 minutes/121°C at 15-pound pressure and then each file was placed in a test tube containing nutrient agar; Group B: Files in this group were socked in an aldehyde-free instrument disinfectant (Cleanmed Instrument from BMS Dental) for 60 minutes (Manufacturer’s recommendations) and then each file was placed in a test tube containing nutrient agar; Group C: Files in this group were immediately placed in the test tubes containing nutrient agar without any sort of sterilization (Control group); Group D: Files in this group were irradiated using a diode laser system (PRIMO Dental Laser System from Medency) for 3 seconds for each surface at 10 watts (980 nm). During the 3-second time, the laser beam was moved along the file length and then the file was rotated to irradiate all surfaces. The files were handled using sterilized tweezers to change the surface of exposure while keeping the laser beam at a 3 cm fixed distance away from the file. After complete irradiation, the shafts of the instruments were removed from the handle utilizing a sterile wire cutter, and each file was placed in a test tube containing nutrient agar.

**Figure 1:**
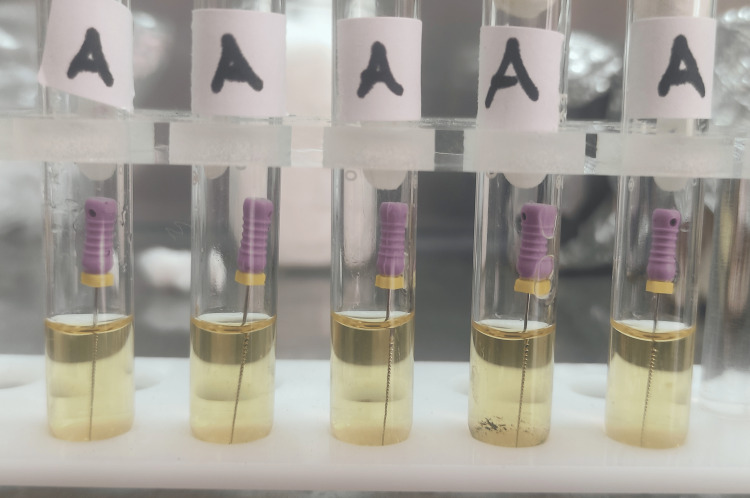
Group A test tubes after 24 hours of incubation. There was no turbidity in any tube.

**Figure 2:**
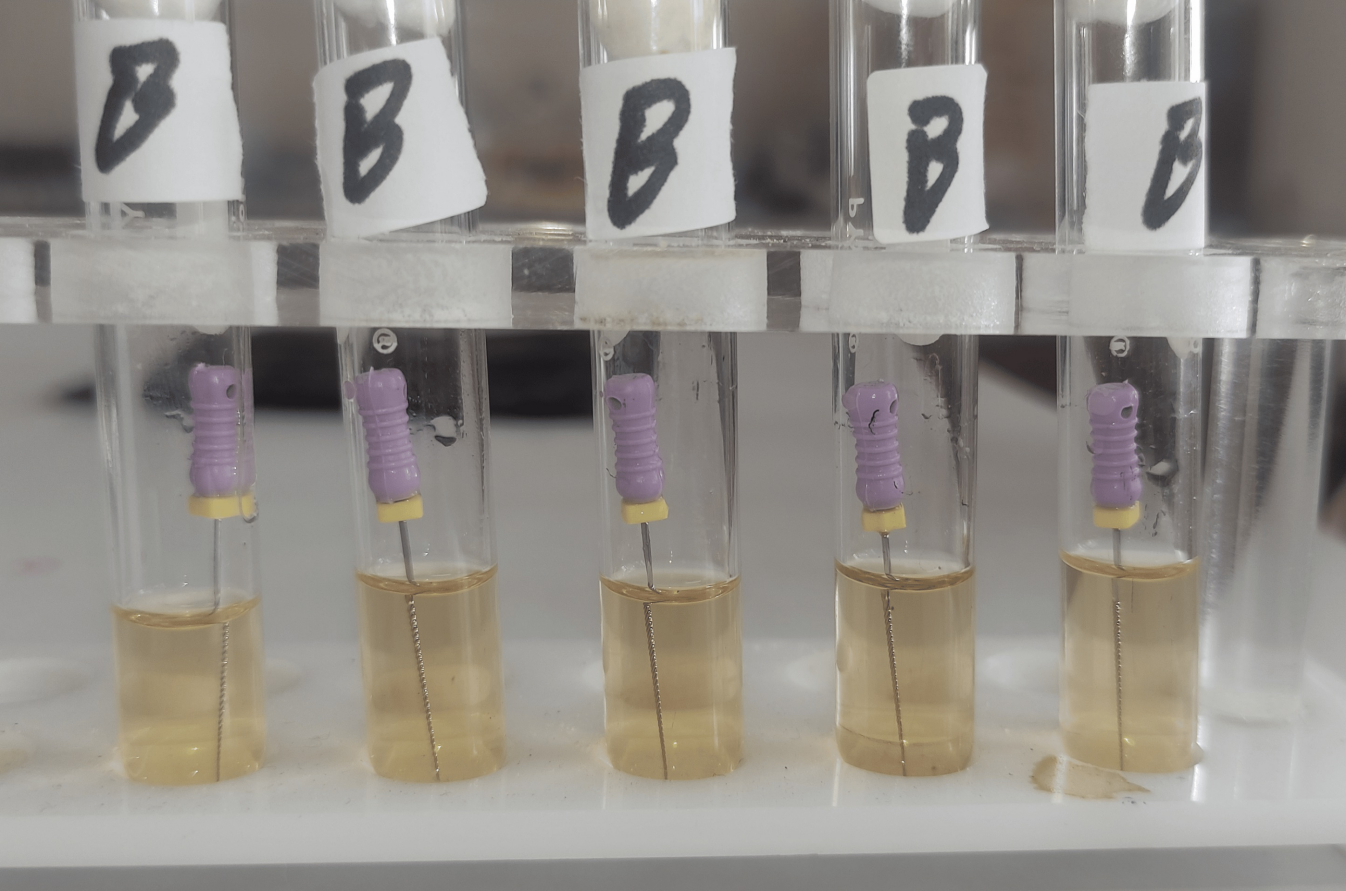
Group B test tubes after 24 hours of incubation. There was no turbidity in any tube.

**Figure 3:**
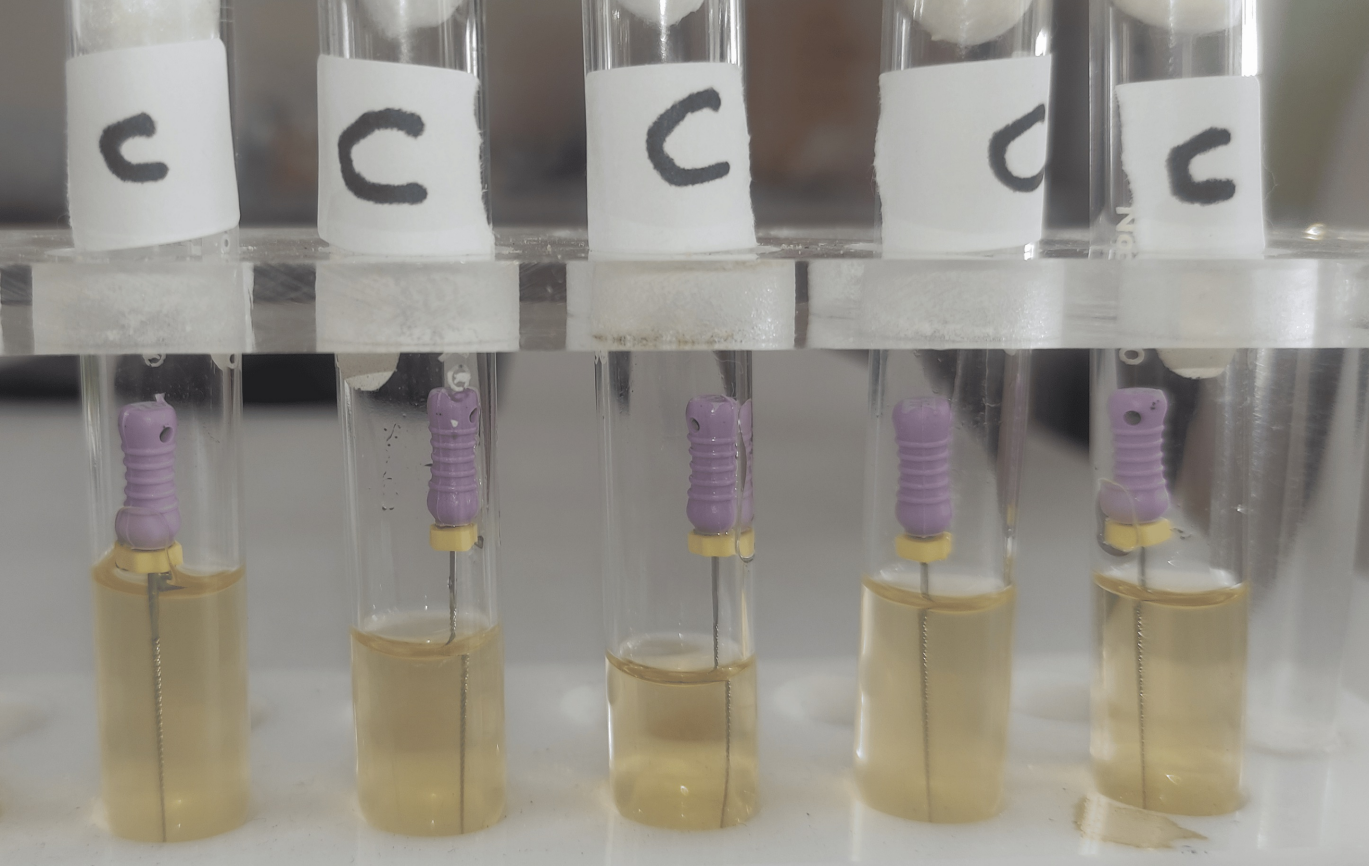
Group C test tubes after 24 hours of incubation. Turbidity was seen in all tubes.

**Figure 4:**
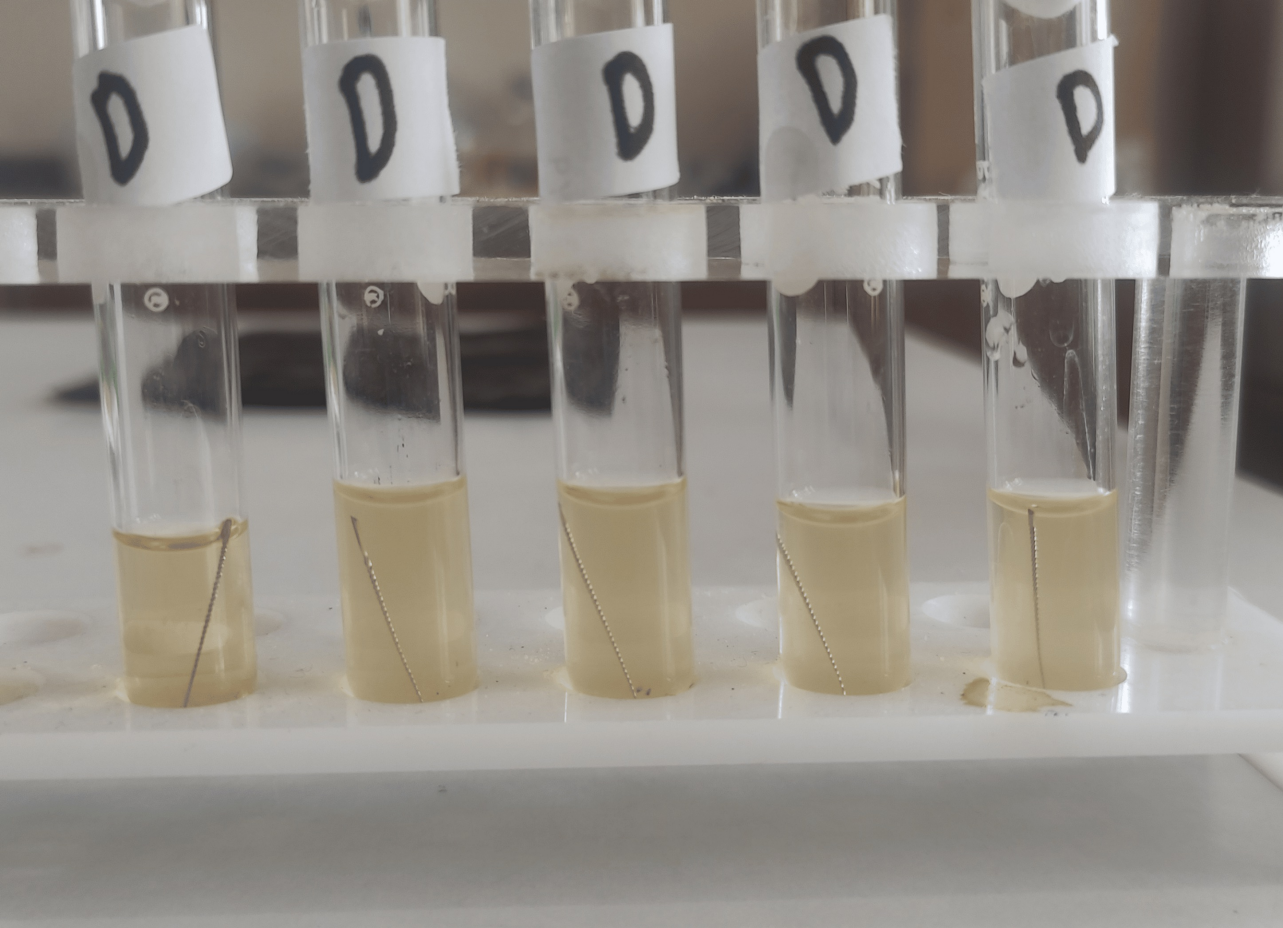
Group D test tubes after 24 hours of incubation. Turbidity was seen in four tubes.

Test tubes containing the files were then incubated at 37°C for 24 hours. The test tubes were then removed from the incubator and each test tube was checked for turbidity. The presence of turbidity in a test tube indicated the presence of *E. faecalis* and that the particular file was not sterilized completely. A sample was taken from each tube to detect bacterial growth using a sterile cotton swab (discarded after one use) and cultured using the Spread Plate technique on plates containing Mueller-Hinton agar. Each tube is assigned a plate. The plates were then incubated for 24 hours at 37°C. The test tubes were then further kept in incubation at 37°C for another 24 hours and the test tubes were removed from the incubator and each test tube was checked again for turbidity (Figures 1-4).

## Results

Bacterial growth on plates

**Figure 5:**
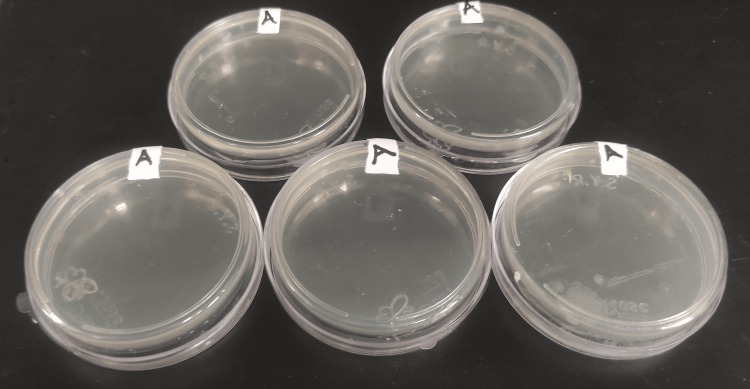
Group A plates after 24 hours of incubation.

**Figure 6:**
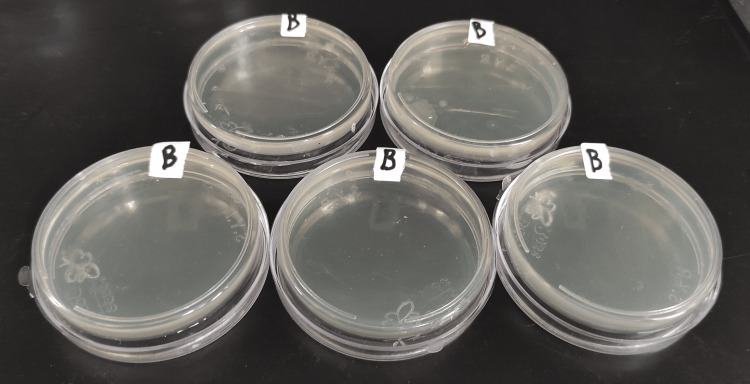
Group B plates after 24 hours of incubation.

**Figure 7:**
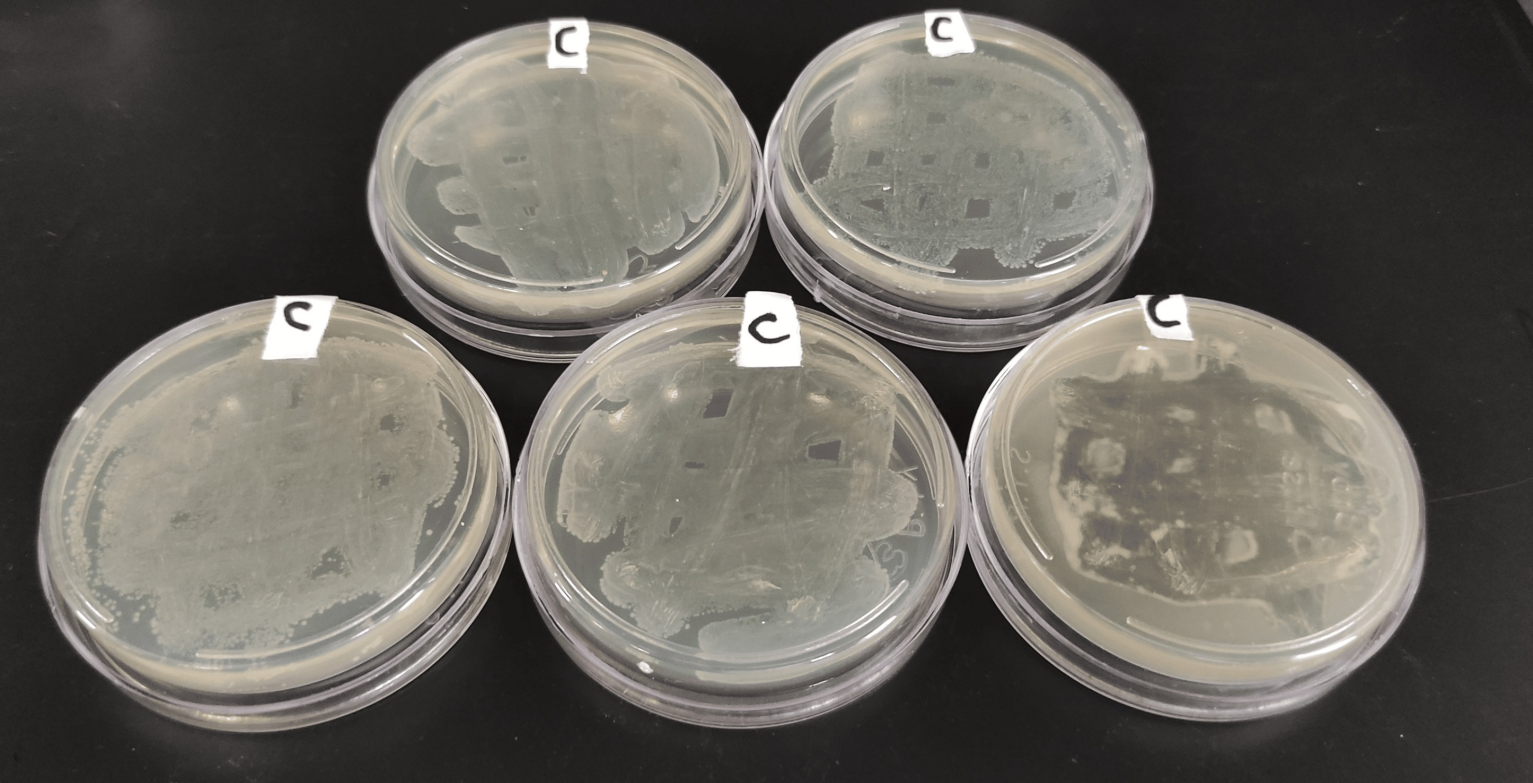
Group C plates after 24 hours of incubation.

**Figure 8:**
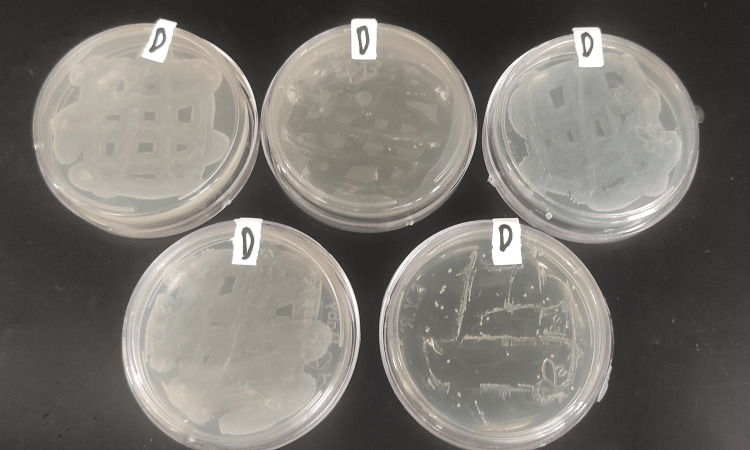
Group D plates after 24 hours of incubation.

After 24 hours of incubation, plates in group A showed no bacterial growth in all plates (Figure 5). Plates in group B showed no bacterial growth in all plates (Figure 6). Plates in group C showed bacterial growth in all plates (Figure 7). Plates in group D showed bacterial growth in all plates (Figure 8; Table 1).

**Table 1:** Bacterial growth on the groups’ plates.

	Bacterial growth				
	Plate 1	Plate 2	Plate 3	Plate 4	Plate 5
Group A	-	-	-	-	-
Group B	-	-	-	-	-
Group C	+	+	+	+	+
Group D	+	+	+	+	+

Turbidity in test tubes

**Figure 9:**
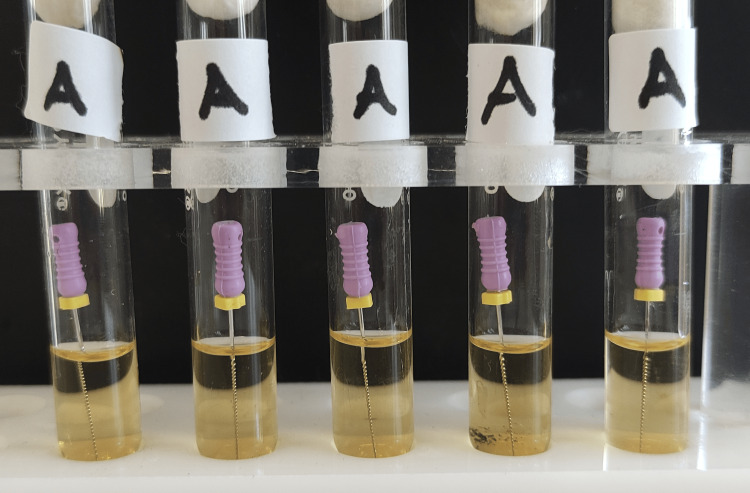
Group A test tubes after 48 hours of incubation.

**Figure 10:**
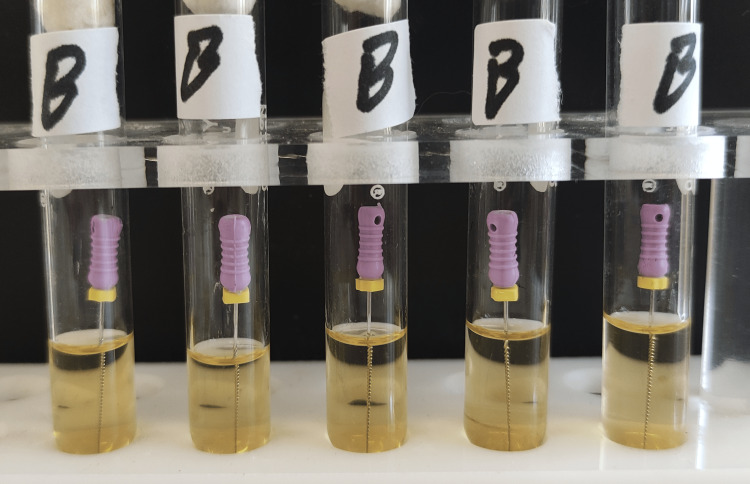
Group B test tubes after 48 hours of incubation.

**Figure 11:**
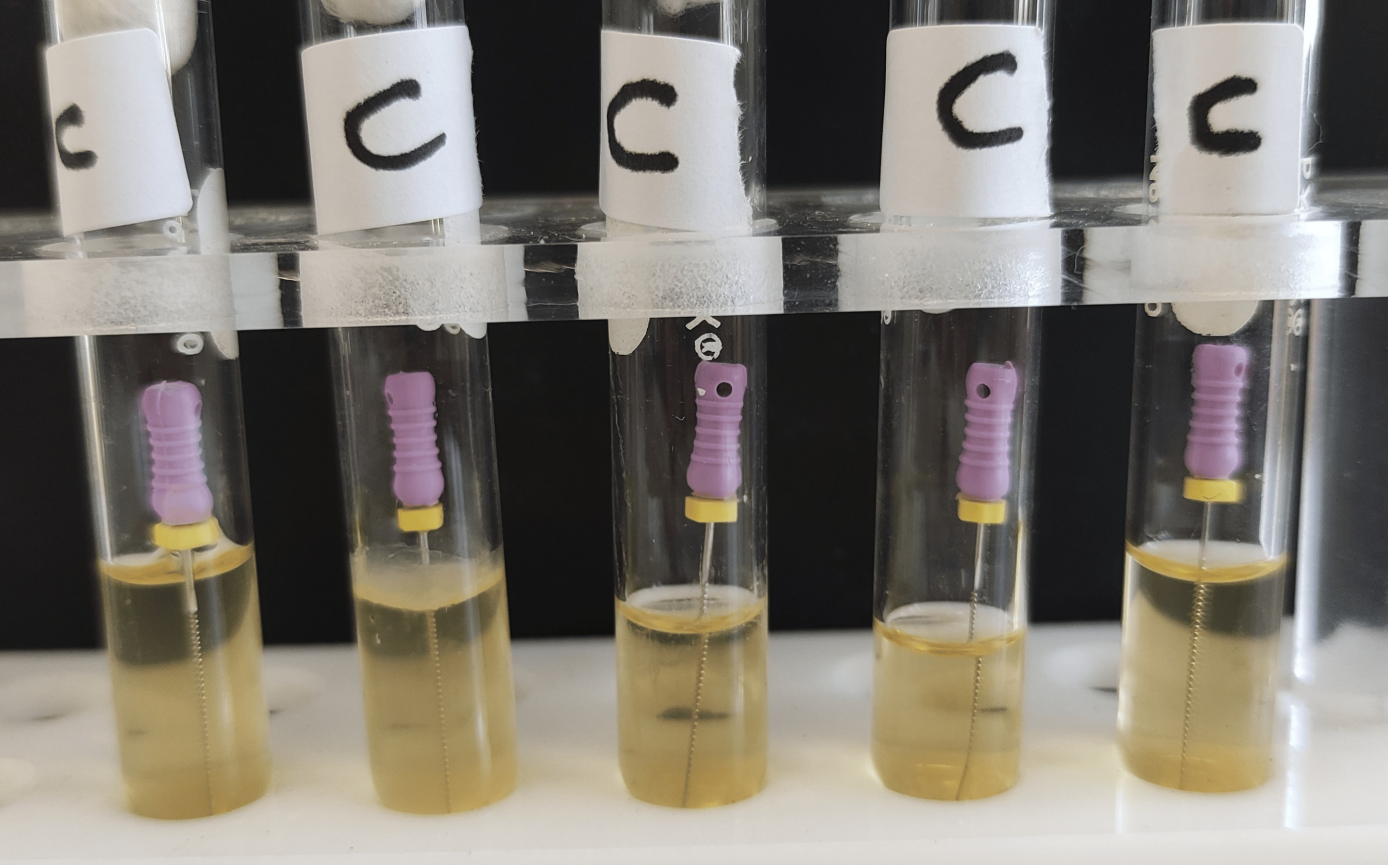
Group C test tubes after 48 hours of incubation.

**Figure 12:**
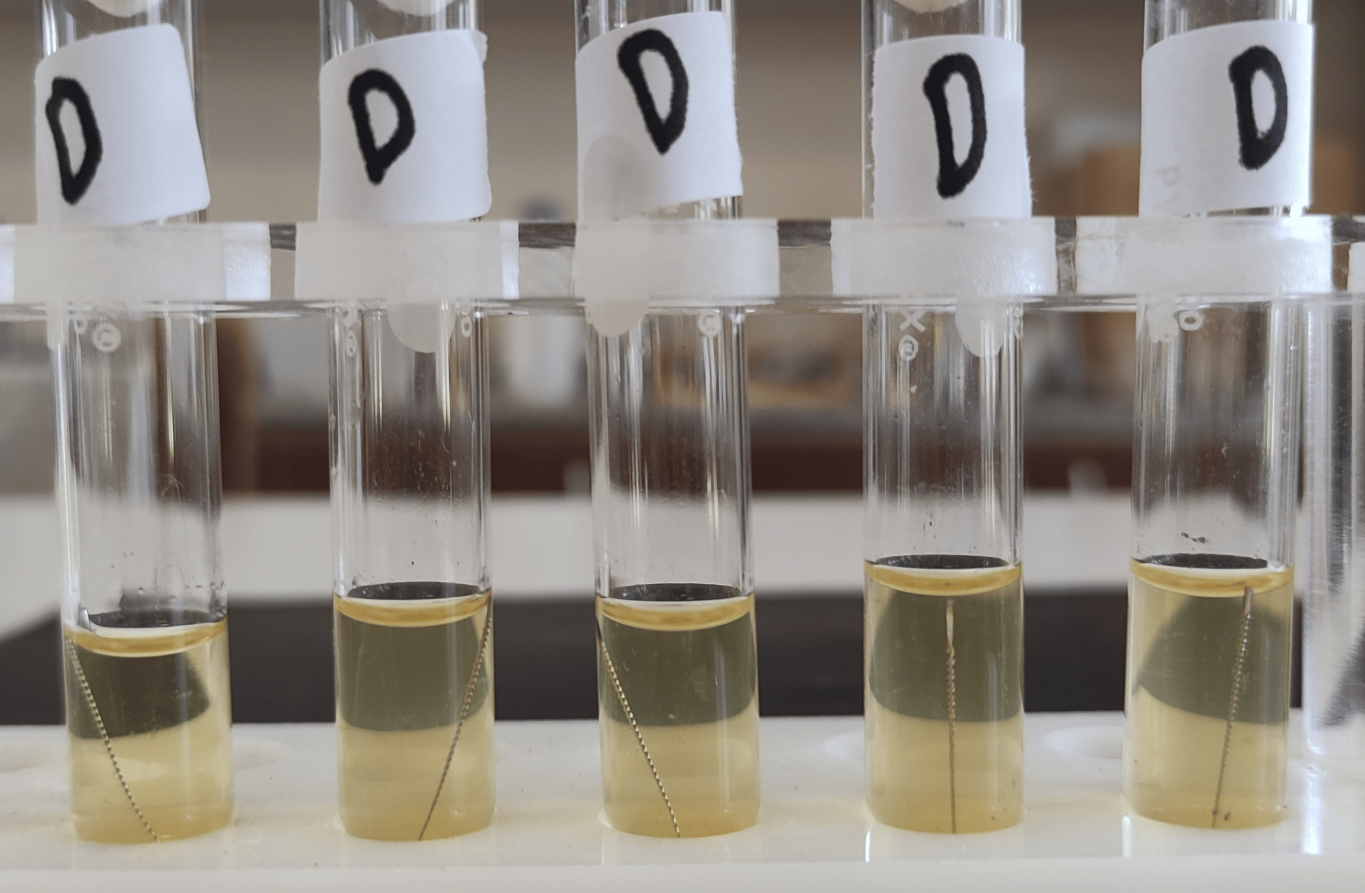
Group D test tubes after 48 hours of incubation.

After 48 hours of incubation, test tubes in group A showed no turbidity in all tubes (Figure 9). Test tubes in group B showed no turbidity in all tubes (Figure 10). Test tubes in group C showed turbidity in all tubes (Figure 11). Test tubes in group D showed turbidity in all tubes (Figure 12; Table 2).

**Table 2:** Presence of turbidity in the groups’ test tubes.

	Presence of turbidity				
	Tube 1	Tube 2	Tube 3	Tube 4	Tube 5
Group A	-	-	-	-	-
Group B	-	-	-	-	-
Group C	+	+	+	+	+
Group D	+	+	+	+	+

## Discussion

Several studies have discovered bacterial contamination in new endodontic files, even when the file packages are sealed and sterilized by the manufacturer, thus all endodontic files should be sterilized before being used even the new unused files [1-3].

*E. faecalis* is commonly isolated from infected root canals, thus it is considered one of the pathogens associated with unsuccessful endodontic treatment. *E. faecalis* can be frequently found in failed endodontic treatment due to its ability to survive an environment that lacks nutrients and in root canals that are highly alkaline after using medicaments for several weeks [9]. Thus, it was used in this study that aims to evaluate the effectiveness of four methods to sterilize endodontic files to be reused without transition of bacteria that can influence the endodontic treatment prognosis.

This study used an aldehyde-free instrument disinfectant that is common in the Syrian market. The use of aldehyde-based disinfectants remains limited due to their toxic properties and risks. It is important to avoid contact with the skin because of the allergic reactions it causes, it is also dangerous to inhale [10]. Additionally, the effectiveness of sterilization with glutaraldehyde (the most used type of aldehyde-based disinfectant) is controversial in the medical literature. Many studies indicated incomplete sterilization while using glutaraldehyde 2.4% [5].

Files in group A showed sterility (by showing no turbidity in test tubes after incubation for 48 hours and no bacterial growth on plates after incubation for 24 hours) after being contaminated with *E. faecalis* and then autoclaved. This result is compatible with the studies done by El-Tayed et al., Venkatasubramanian et al., Al-Jamell et al., Yenni et al., and Raju et al., which showed that autoclave is an effective method for sterilizing endodontic files, and dental burs [5-7,11-13].

Files in group B showed sterility (by showing no turbidity in test tubes after incubation for 48 hours and no bacterial growth on plates after incubation for 24 hours) after being contaminated with *E. faecalis* and then socked in the chemical instrument disinfectant for 60 minutes. This result is compatible with the study done by Yenni et al., which used another commercial type of chemical disinfectant, but similar in composition to the disinfectant used in this study [11]. Cleanmed Instrument from BMS Dental is a disinfectant that contains quaternary ammonium compounds which have antimicrobial applications; thus, they are utilized in a wide variety of products. The bactericidal action of the quaternary has been attributed to the inactivation of energy-producing enzymes, denaturation of essential cell proteins, and disruption of the cell membrane [14].

Files in group C showed turbidity in test tubes after incubation for 48 hours and bacterial growth on plates after incubation for 24 hours after being contaminated with *E. faecalis*, thus this group which acted as a control group, confirmed the successful contamination of files with *E. faecalis* colonies.

Files in group D showed turbidity in test tubes after incubation for 48 hours and bacterial growth on plates after incubation for 24 hours after being contaminated with *E. faecalis* and then irradiated with a diode laser system. This result is compatible with the studies done by Al-Jamell et al. (which found that autoclave achieved full sterilization, whereas using diode laser or CO_2_ laser could not achieve full sterilization with a slight superiority of CO_2_ laser over diode laser) [7]. Ameer et al. found that an autoclave achieved full sterilization whereas using a diode laser could not achieve full sterilization) [8].

Several studies showed the effectiveness of sterilizing endodontic files with CO_2_ laser and some of these studies even compared its results with autoclave [6,12], However in this study we used a diode laser as it is far more common to see in dental clinics compared to CO_2_ laser.

On account of the fact that this study was performed on a specific bacterium, the results do not mean that the sterilizing method is effective. It simply means that the method is effective against the specific bacteria, and thus further study on a variety of microorganisms should be conducted in order to consider this method effective for sterilizing files.

## Conclusions

Within the limits of this study, the autoclave and Cleanmed Instrument were capable of eliminating *E. faecalis*. Irradiation with a diode laser system was not capable of eliminating this bacterium.
